# Hyaluronate-Thiol Passivation Enhances Gold Nanoparticle Peritumoral Distribution When Administered Intratumorally in Lung Cancer

**DOI:** 10.3390/biomedicines9111561

**Published:** 2021-10-28

**Authors:** Rossana Terracciano, Yareli Carcamo-Bahena, E. Brian Butler, Danilo Demarchi, Alessandro Grattoni, Carly S. Filgueira

**Affiliations:** 1 Department of Nanomedicine, Houston Methodist Research Institute, Houston, TX 77030, USA; rterracciano@houstonmethodist.org (R.T.); ycarcamo@houstonmethodist.org (Y.C.-B.); agrattoni@houstonmethodist.org (A.G.); 2 Department of Electronics and Telecommunications, Politecnico di Torino, 10129 Torino, Italy; danilo.demarchi@polito.it; 3 Department of Radiation Oncology, Houston Methodist Research Institute, Houston, TX 77030, USA; ebutler@houstonmethodist.org; 4 Department of Surgery, Houston Methodist Research Institute, Houston, TX 77030, USA; 5 Department of Cardiovascular Surgery, Houston Methodist Research Institute, Houston, TX 77030, USA

**Keywords:** gold nanoparticles, hyaluronate-thiol, in vitro, in vivo, peritumoral, cancer, cytotoxicity, ICP-OES, biodistribution

## Abstract

Biofouling is the unwanted adsorption of cells, proteins, or intracellular and extracellular biomolecules that can spontaneously occur on the surface of metal nanocomplexes. It represents a major issue in bioinorganic chemistry because it leads to the creation of a protein corona, which can destabilize a colloidal solution and result in undesired macrophage-driven clearance, consequently causing failed delivery of a targeted drug cargo. Hyaluronic acid (HA) is a bioactive, natural mucopolysaccharide with excellent antifouling properties, arising from its hydrophilic and polyanionic characteristics in physiological environments which prevent opsonization. In this study, hyaluronate-thiol (HA-SH) (MW 10 kDa) was used to surface-passivate gold nanoparticles (GNPs) synthesized using a citrate reduction method. HA functionalized GNP complexes (HA-GNPs) were characterized using absorption spectroscopy, scanning electron microscopy, zeta potential, and dynamic light scattering. GNP cellular uptake and potential dose-dependent cytotoxic effects due to treatment were evaluated in vitro in HeLa cells using inductively coupled plasma—optical emission spectrometry (ICP-OES) and trypan blue and MTT assays. Further, we quantified the in vivo biodistribution of intratumorally injected HA functionalized GNPs in Lewis Lung carcinoma (LLC) solid tumors grown on the flank of C57BL/6 mice and compared localization and retention with nascent particles. Our results reveal that HA-GNPs show overall greater peritumoral distribution (** *p* < 0.005, 3 days post-intratumoral injection) than citrate-GNPs with reduced biodistribution in off-target organs. This property represents an advantageous step forward in localized delivery of metal nano-complexes to the infiltrative region of a tumor, which may improve the application of nanomedicine in the diagnosis and treatment of cancer.

## 1. Introduction

Tumor targetability and site-specific release are considered the most critical factors in cancer diagnostics and therapy. To date, local chemotherapeutic and immunotherapeutic drug delivery has demonstrated superior efficacy and safety compared to systemic administration in murine models of cancer [1,2,3]. When a drug is retained within the tumor microenvironment (TME) at high concentrations, its local therapeutic effect is enhanced and any off-target side effects due to systemic exposure are minimized [4,5,6]. Unfortunately, local intratumoral injection of free anti-cancer agents into solid tumors can be compromised by their leaky vasculature [7] and elevated interstitial fluid pressure [8], which leads to rapid escape of the drug from the tumor confinement and can contribute to reduced sensitivity to therapy [9,10] or primary resistance [11].

To overcome these challenges, more investigations are needed to identify novel successful strategies for site-directed drug localization within a solid tumor to target and understand the body’s response toward the introduced material [12]. In this context, gold nanoparticles (GNPs) have been investigated as anti-cancer drug cargo carriers, whereby their anti-angiogenic and photothermal properties can be used to potentialize drug action [13,14], and their diffusive nature exploited for multimodal synergistic diagnosis and therapy [15,16]. In addition, GNPs are of great interest in the field of nanotherapeutics because they are considered nontoxic, hydrophilic, have tailorable charge and size, and have modifiable surface chemistry. Further, metallic nanoparticles provide therapeutic and diagnostic advantages that can be used in conjunction with cargo delivery, such as highly localized photothermal heating responses [17], enhanced contrast proprieties for computed tomography and photoacoustic imaging [18], and they can act as molecular sensors [19]. However, uniform intratumoral distribution of GNPs with tumor site-specificity and retained accumulation has not yet been achieved according to some recent pre-clinical studies [20,21].

Evaluating the tumoral distribution of a drug or particle-drug conjugate is an important variable that is often neglected, as most drugs are assumed to distribute homogeneously into a tumor [22]. In previous studies, we demonstrate that GNP cellular uptake [23,24], as well as intratumoral distribution and retention [25,26], is strongly influenced by surface chemistry. We have shown that when GNPs are surface passivated with a bovine serum albumin (BSA) protein layer, internalization of the particles into cancer cells occurs through larger vesicle formation with reduced gold content per cell, and when injected intratumorally in a murine lung cancer model, the BSA passivated particles diffuse more overtime throughout the tumor tissue [25]. BSA modification shields the metal core and prevents unwanted non-specific adsorption of other agents onto the particles. However, while BSA is inexpensive, easily accessible, and provides a model substrate, its passivation is non-uniform, there is a possibility for other proteins to overcoat the BSA, and it does not bind with high specificity to a receptor target expressed on cancer cells [27]. Compared to passive targeting, active targeting of GNPs is a promising strategy for nanoparticle-mediated drug delivery since it relies on a biological interaction between ligands on the GNP surface and receptors on the targeted cancer cell [28]. Nevertheless, when the GNP surface is functionalized with targeting ligands, such as proteins, peptides, polysaccharides, apatmers, or small molecules, the consequences of the corona must be considered. In fact, non-specific interactions between engineered nanomaterials and the biological microenvironment of the tumor lead to unwanted adsorption of molecules which can mask or displace the conjugated ligands on the GNP surface. Furthermore, uncontrolled or unmonitored changes in nanoscale structure, chemical composition, or molecular conformation may dramatically affect physiological response to a pharmaceutical or nanoscale device [29]. In a biological context, this effect is identified as *biofouling,* and it leads to the loss of targeting affinity of GNPs or, in a worst-case scenario, redirects the complexes to other undesired sites [30,31]. Therefore, innovative anti-biofouling strategies must be investigated when a novel diagnostic and/or therapeutic approach is proposed for cancer applications [32,33,34].

In this study, we investigate the effects of GNP surface passivation with hyaluronate on particle intratumoral distribution in solid lung carcinoma. Hyaluronate or hyaluronic acid (HA) is a negatively charged, non-sulfated, linear biopolymer composed of alternatingly linked saccharide units of glucuronic acid and N-acetylglucosamine [35]. HA is a naturally produced biocompatible material since it is an essential component of the extracellular matrix [36]. It is found abundantly in skin [37], cartilage [38], synovial fluid [39], and interstitial fluids [40], and is naturally biodegradable. To date, it is used in clinical settings as an injectable hydrogel [41] and biological scaffold [42] in different tissue engineering applications, such as for dermatological fillers and osteoarthritis treatment [43]. 

HA is the principle ligand of the cluster of differentiation 44 (CD44) receptor, which is a glycoprotein expressed at low levels on the surface of hematopoietic and epithelial mammalian cells [44], but overexpressed in many tumor cells, including several non-small cell lung cancers [45]. Moreover, HA is a versatile compound since it offers multiple sites for chemical modification, and it has been previously reported to prevent undesired protein corona formation on the nanomaterial’s surface [46]. In fact, if chemically modified with a thiol (-SH) functional group, forming a hyaluronate-thiol complex (HA-SH), it can covalently bind to the surface of the GNPs, improving upon their stealth behavior as well as their targeting specificity for CD44 receptors [47]. 

The aforementioned characteristics as well as its high viscoelasticity and potential for chemical modifications to the backbone structure all make HA an ideal candidate for anti-cancer applications involving engineered gold-based nanomaterials [48]. Many research works present different synthesis processes of HA-GNP complexes [49], as well as their in vitro testing as drug carriers for photothermal cytotoxic drug release in cancer cells [50]. We identified certain studies involving pre-clinical cancer models; however, the HA coated GNPs are only administered systemically, and biodistribution is not evaluated [51,52,53,54]. Only a few studies have assessed HA-GNP distribution in various pre-clinical models, such as the porcine eye, as HA is a widely used excipient in ocular drug delivery, and rodent skin, as HA has the ability to stimulate fibrin development in the wound healing process. For instance, Apaolaza et al. [55] immobilized low molecular weight HA (5 kDa) on the surface of GNPs and visually observed that HA passivation promoted enhanced particle distribution across the vitreous matrix and ocular tissues after local injection in ex vivo porcine eyes. Sonntag et al. [56] also conjugated thiolated HA to GNPs of various sizes (5, 60, 80, and 120 nm) and quantified their distribution in the anterior chamber of the eye in an ex vivo porcine model. The authors found that the HA coating prevented aggregation of NPs inside the trabecular meshwork and yielded reduced gold content in off-target tissues in the anterior eye, such as the cornea, lens, iris, and ciliary body. Mendes et al. [57] performed a visual analysis of the wound area contraction in the skin of rats after daily topical application of GNPs electrostatically functionalized with HA followed by laser irradiation for seven days, demonstrating a decrease in inflammatory infiltrate and an increase in wound contraction post-photobiomodulation treatment. While these studies all illustrate that HA coated GNPs can show improved tissue targeting and efficacy, to our knowledge there is no single pre-clinical study which evaluates and quantifies the intratumoral distribution of HA-GNPs. Therefore, in this work, after confirming successful surface modification and demonstrating their anti-biofouling properties in plasma and cancerous cells, we exploit elemental analysis to quantify site-specific HA-GNP accumulation in different tumor regions following local particle administration in vivo. As there are no pre-clinical studies which investigate or present changes in intratumoral distribution patterns as a result of HA passivation, the novelty of this work is to provide evidence that gold nanoparticles passivated with HA show greater peritumoral accumulation. As the TME is notoriously complex and solid tumors can be considered “abnormal organs” composed of multiple cell types [58], identifying nanoparticle localization within particular tumor zones could be used to improve drug delivery to specific cell types that direct and influence cancer growth and progression.

## 2. Materials and Methods

### 2.1. Gold Nanoparticles Synthesis 

GNPs synthesis was achieved by combining 7 mL of 0.033 M gold (III) chloride (Sigma, St. Louis, MO, USA, 379948) and 4.8 mL of 0.039 M aqueous citrate (Sigma, St. Louis, MO, USA, C3674) into an Erlenmeyer flask containing 600 mL of boiling Milli-Q water, following a previously reported approach [23]. The dark-red colloidal solution obtained from this process was allowed to equilibrate to room temperature for 24 h. Once cooled, the pH of the solution was measured using an Accumet AE150 benchtop pH meter (Fisher Scientific, Hampton, NH, USA, 13-636-AE153). After synthesis, the GNPs have a native acidic pH due to their stabilization with citric acid (pH = 3.6). We previously demonstrated that in this condition, the particles lose their stability when immersed in biological media [25]. To increase particle stability, the pH of the solution was adjusted to 6.0 through dropwise addition of a 1 M NaOH solution. The GNPs were concentrated and washed by centrifuging 15 mL of the solution (pH = 6.0) at 1500× *g* for 5 min in Amicon Ultra-15 100K filters (Sigma-Aldrich, St. Louis, MO, USA, UFC910008) using an Eppendorf Centrifuge 5810R (Hamburg, Germany), and the pellet was then resuspended in 1 mL Milli-Q water at pH = 6.0. This process was performed to remove any excess citrate that may impair the hyaluronate-thiol surface functionalization. Prior to centrifugation, the stability of the colloidal gold was assessed through visual inspection of the solution along with UV-VIS spectroscopy (see Section 2.3.1). GNPs were stored at room temperature. Particle reproducibility was tested synthetizing three different batches of HA-GNPs.

### 2.2. Surface Passivation of the Gold Nanoparticles with Hyaluronate-Thiol

For surface passivation of the GNPs with hyaluronate-thiol, HA-SH powder 10 kDa (Nanosoft Polymers, Winston-Salem, NC, USA, Lot#246561003, PDI ~ 1.5) was used in this study, following the protocol described by Lee et al. [59]. HA-SH solution (5 mg/mL) was prepared by mixing 5 mg of HA-SH powder in 1 mL Milli-Q water until complete dissolution. Next, 0.004 mL of the HA-SH solution was added to 1 mL of the washed GNPs (obtained as described in Section 2.1), and the obtained solution was vortexed to allow for a more complete and homogeneous sample. UV-VIS spectra were acquired after 1 and 24 h to check particle stability and assess changes in the surface plasmon resonance (SPR). The solution was stored at room temperature.

### 2.3. GNP Characterization

After synthesis, HA-GNP stability was assessed though visual inspection (whereby no macro-aggregation or flocculation was observed, as well as no changes in the dark-red color of the solution). The optical properties as well as the morphology of the HA-GNPs were evaluated using UV-VIS spectroscopy and scanning electron microscopy (SEM). Particle morphology was assessed using a FEI Nova NanoSEM 230 (FEI Co., Hillsboro, OR, USA). Changes in surface chemistry and opsonization/antifouling properties were evaluated with dynamic light scattering and ζ-potential. 

#### 2.3.1. Ultraviolet-Visible (UV-VIS) Spectroscopy

For all the samples, absorbance spectra were obtained on a Beckman-Coulter UV-VIS (200–1000 nm) spectrophotometer (DU 730, Beckman Coulter, Inc., Brea, CA, USA). Low concentrations of sample (<1 mg/mL) were used in 1 mL volumes by combining a 1:1 ratio of particles in Milli-Q at pH 6.0 with either PBS (1× phosphate buffered saline at pH 7.4, Thermo Fisher Scientific, Waltham, MA, USA, SH30256FS) or plasma (at a measured pH 7.4), following a blank measurement with Milli-Q water. 

#### 2.3.2. Dynamic Light Scattering (DLS) and ζ-Potential

To confirm passivation, assess surface charge, and test colloidal stability as well as the antifouling properties of the GNPs in a biological medium, dynamic light scattering (DLS) and ζ-potential measurements were conducted on the samples prepared as described in Section 2.3.1. Particle size (hydrodynamic diameter), polydispersity index (PDI), and surface charge (ζ-potential) after synthesis and passivation with HA was performed in Milli-Q water, PBS, and blood plasma using a Zetasizer Nano ZS (Malvern Panalytical, Westborough, MA, USA). Particle size was measured using a standard cubic cuvette, while surface charge (ζ-potential) was obtained using a dip cell ZEN1002. In both analyses, low sample concentration (<1 mg/mL) was used in a 1 mL volume. All of the measurements were conducted at room temperature. Whole blood from healthy porcine (male castrated domestic pigs, ~66 kg, Oak Hill Genetics, Ewing, IL, USA) following an approved protocol by the Institutional Animal Care and Use Committee (IACUC) at the Houston Methodist Research Institute (IS00005819 approved 26 March 2021) was collected in K_2_ EDTA coated blood collection tubes (BD Vacutainer, Franklin Lakes, NJ, USA, 367861) and centrifuged for at least 15 min at 1200× *g*. 

### 2.4. In Vitro Assessment of Cytotoxicity and GNP Uptake

#### 2.4.1. Cell Line and Passaging

HeLa cells derived from human cervical cancer (ATCC^®^, Manassas, VA, USA, ATCC^®^ CCL-2) were grown in T-75 flasks using Eagle’s minimum essential medium (EMEM) supplemented with 10% fetal bovine serum (FBS, USDA approved, ATCC^®^, Manassas, VA, USA) and incubated at 37 °C and 5% humidity in a HERAcell 150i CO2 incubator (Thermo Fisher Scientific, Waltham, MA, USA). To expand and passage the cell line, the cells were first washed with sterile 1x PBS and then 0.25% trypsin-0.53 mM EDTA solution (Thermo Fisher Scientific, Waltham, MA, USA, 25-200-056) was added to the flask in a sterile field to dissociate the cells from the flask and each other. After placing them in the cell culture incubator for 5 min, the cell suspension was neutralized with complete growth media. The suspension was then centrifuged at 130× *g* for 5 min, the supernatant discarded, and the pellet containing living cells resuspended in at least 1 mL of complete growth media. Final cell concentration was obtained using a Countess™ II FL Automated Cell Counter (Invitrogen, Carlsbad, CA, USA).

#### 2.4.2. MTT Assay for Cell Viability Due to Metabolic Activity

Viability related to cell proliferation and metabolic activity after nanoparticle treatment was evaluated through reduction of 3-(4,5-dimethylthiazolyl-2)-2,5-diphenyltetrazolium bromide (MTT) to the blue product formazan, assessed using spectroscopy. HeLa cells were seeded into 96-well plates (Corning™ Costar™ 96-Well, Cell Culture-Treated, Flat-Bottom Microplate, Thermo Fisher Scientific, Waltham, MA, USA, 15250061) at a concentration density of 1 × 10^5^ cells/mL and allowed to incubate for 24 h. It has been previously shown that the administration method of coated GNPs in cell culture can affect particle interaction with cells, such as macrophages, while colloidal stability is independent of the administration method, and pre-mixing the GNP solution in complete cell culture media prior to cell exposure is the best method tested to date [60]. Therefore, after the cell attachment incubation period, conditioned media was replaced with fresh complete media in which the GNP treatment was pre-mixed to avoid unwanted particle deposition. Triplicate wells containing only media and each of the GNP test treatments (wells without cells) were used as controls to remove any background signal. Two different GNP treatment concentrations were investigated: 15 μg [Au]/mL per well and 50 μg [Au]/mL per well. After 24 h of HeLa cell incubation with each of the GNP treatments, 10 µL of MTT Reagent (ATCC^®^, American Type Culture Collection, Manassas, VA, USA) was added and mixed to each well, including the wells without cells, and the samples placed back in the cell culture incubator for approximately 2 h. Once the purple precipitate was clearly visible under the microscope (Nikon ECLIPSE Ts2 Inverted Microscope, Nikon Instruments Inc., Melville, NY, USA), 100 µL of Detergent Reagent (ATCC^®^, American Type Culture Collection, Manassas, VA, USA) was added at room temperature to each well to dissolve the formazan and create a homogeneous dark-colored solution. Absorbance of this solution in each well was measured using a Synergy™ H4 Hybrid Microplate Reader (BioTek Instruments, Inc., Winooski, VT, USA) at 570 nm and 690 nm. 

#### 2.4.3. Trypan Blue Assay for Cell Viability

Viability related to cell membrane integrity was performed using a Trypan blue assay. Trypan blue enters the cells with compromised membranes and stains the dead cells blue. The total number of viable and dead cells was obtained after each GNP treatment (citrate-GNPs or HA-GNPs) and compared with a negative control (untreated cells). To perform this assay, HeLa cells were seeded in 6-well plates (Corning™ Costar™ Clear 6-Well Plate, Thermo Fisher Scientific, Waltham, MA, USA, 07201588) at a concentration density of 1 × 10^5^ cells/mL per well and placed in the cell culture incubator for attachment and growth for 24 h. The media was then replaced with fresh complete media in which the GNP treatments (50 μg [Au]/mL per well) were pre-mixed to avoid unwanted particle deposition. Each treatment (citrate-GNPs at 50 μg [Au]/mL per well, HA-GNPs at 50 μg [Au]/mL per well, or media replacement without GNP) was administered to triplicate wells, and the samples were placed in the cell culture incubator for 24 h. After incubation, 0.4% Trypan Blue Solution (Thermo Fisher Scientific, Waltham, MA, USA, 15250061) was used to differentiate between living and dead cells (1% of the cell volume collected from each well was evaluated) and the cells counted using a Countess™ II FL Automated Cell Counter (Invitrogen, Carlsbad, CA, USA). The pellet (99% of the cell volume collected from each well) was used for inductively coupled plasma optical emission spectrometry (ICP-OES) analysis as described in Section 2.4.4.

#### 2.4.4. Elemental Analysis to Quantify Intracellular GNP Uptake

Inductively coupled plasma optical emission spectrometry (ICP-OES) was used for all of the elemental analyses performed in this study. Standard curves for calibration of Au content were created using seven different standard concentrations: 100 μg [Au]/L, 250 μg [Au]/L, 500 μg [Au]/L, 1000 μg [Au]/L, 2500 μg [Au]/L, 5000 μg [Au]/L, and 10000 μg [Au]/L. Each standard was prepared by serial diluting a gold standard (Au 1000 µg/mL, Perkin Elmer, Waltham, MA, USA, N9303759) in an acidic solution containing 10% trace metal grade hydrochloric acid (HCl, Thermo Fisher Scientific, Waltham, MA, USA) and 1% trace metal grade nitric acid (HNO_3_, Thermo Fisher Scientific, Waltham, MA, USA). 

To quantify the cellular uptake of the HA-GNPs and compare with the uptake of the native particles, cell pellets (as described in Section 2.4.3) were digested in a chemical fume hood using a 1 mL solution of aqua regia (1:3 nitric acid to hydrochloric acid) for 1 h and then diluted in acidic solution (10% HCl, 1% HNO_3_). To avoid any clogs in the tubing systems of the ICP-OES hardware, each sample was filtered prior to analysis using 0.6 μm filters (MilliporeSigma™, Burlington, MA, USA, Steriflip Quick Release-GP Sterile Vacuum Filtration System). All measurements were performed on triplicate samples using a Varian Agilent 720-es ICP spectrometer (Agilent, Santa Clara, CA, USA), and the results were obtained by averaging the signal from two gold emission lines (242.794 nm and 267.594 nm) using the ICP Expert II software. The gold content found in each pellet was normalized to the total number of counted cells (dead and alive). With this normalization, we assume that nanoparticle internalization is homogenous across the cells.

### 2.5. In Vivo Biodistribution Analysis 

#### 2.5.1. Animal Model of Lung Cancer

Six-week-old female C57BL/6 mice (*n* = 20) were used in this study to evaluate and quantify site-specific diffusion of the GNPs as a function of their surface chemistry. The research protocol was granted Institutional Animal Care and Use Committee (IACUC) approval (protocol # IS00005178 approved 6 May 2019) at the Houston Methodist Research Institute. The animals were purchased from Taconic Biosciences (Rensselaer, NY, USA). Female mice were chosen for study because while both naive male and female C57BL/6 mice do not present lung function differences at baseline [61], tumors grow more rapidly in female C57BL/6 mice [62]. In addition, to date, worldwide statistics indicate biological sex differences in human lung cancer, with higher lung cancer incidence rates for women [63]. A Lewis lung carcinoma (LLC) cell line was used in this study as a murine model of non-small cell lung cancer (NSCLC), since it is highly tumorigenic and provides a reproducible syngeneic model for lung cancer in the C57BL mouse [64,65,66].

#### 2.5.2. Experimental Timeline

Under sedation, all mice (*n* = 20, average weight of 18.8 ± 1.3 g) received manual subcutaneous injection of 2 × 10^6^ LLC cells into their right flank. Tumors were palpable 4–5 days after cell injection, and after 10 days, tumor volumes reached an average of ~100 mm^3^. 10 days post tumor cell inoculation, GNPs (citrate-GNPs: 50 μL, 10 mg/mL, *n* = 10 or HA-GNPs: 50 μL, 10 mg/mL, *n* = 10) were intratumorally injected using insulin syringes (BD U 100 Insulin Syringe Micro Fine Needle 28G, Becton, Dickinson and Company, Franklin Lakes, NJ, USA, 329461) with a syringe pump (KD Scientific Inc., Holliston, MA, USA, Model 100) set at a slow rate (0.43 μL/s) and 50 μL dispense volume. We previously demonstrated that automatic injections in small rodents can reduce the variability and error introduced by manual injections of nanoparticles [25]. In addition, we consistently injected the particles in the same region of the tumor (the core) for each animal. Mice weight and health conditions (Figure S1A) were monitored daily, ensuring adequate nutrients (food and water ad lib.) and living conditions (clean cages, enrichment). Tumor volumes were also measured daily using a digital caliper (McMaster-Carr, Elmhurst, IL, USA, 2340A11) (Figure S1B). All animal procedures involving injections were performed by anesthetizing the mice with isoflurane. The animals were sacrificed three days (*n* = 5 for each group) and six days (*n* = 5 for each group) post-intratumoral injection of each GNP treatment. Tumors were excised, weighed ex vivo, flash frozen in liquid nitrogen, and stored at −80 °C for further analysis (see Section 2.5.3.). During the necropsy, pictures of the tumor orientation and injection site were taken to keep track of the relative spatial location in reference to the mouse body, allowing for the creation of orientation maps during analysis. Blood was collected by cardiac puncture immediately after death, and the brain, heart, lung, liver, spleen, and kidneys were harvested and digested for elemental analysis. For sample digestion, each organ tissue or blood sample was immersed in 2 mL of fresh aqua regia, heated at 60 °C for 1 h until complete digestion, and resuspended in a 10 mL acid solution composed of 1% nitric acid and 10% hydrochloric acid. Each sample was filtered using 0.22 μm filters (MilliporeSigma™, Burlington, MA, USA, Z359904) prior to ICP-OES measurement. Gold concentration was determined following the same protocol as described in Section 2.4.4.

#### 2.5.3. Site-Specific Intratumoral Distribution of GNP: Elemental Analysis

Frozen tumors were sectioned using a surgical blade to divide the medial and lateral sides in reference to the mouse body. All the dissection procedures were performed by the same investigator (R.T.) for consistency. From each side (medial and lateral), the core was separated from the tumor periphery with a surgical blade. Hypothesizing the tumor volume as a sphere of radius R in a spherical coordinate system, we define the *core* as an internal sphere with Euclidean distance from the origin O to R/2, and the *periphery* as the external shell of the tumor with Euclidean distance from R/2 to R. We also established that the origin O coincides the site of injection (see Section 2.5.1). Therefore, from each tumor, 4 sections were obtained, representing the (i) medial peritumor (MP), (ii) medial core (MC), (iii) lateral core (LC), and (iv) lateral peritumor (LP) as described in Section 3.3. The procedure was performed while the sample remained frozen to avoid any unwanted dispersion of fluids and to increase the precision of the dissection procedure. Each section was digested for elemental analysis to determine any site-specific accumulation of gold dependent on the surface chemistry. For sample digestion, tissues were immersed in 2 mL of fresh aqua regia, heated at 60 °C for 1 h until complete digestion, and resuspended in acid solution composed of 1% nitric acid and 10% hydrochloric acid. Each sample was filtered using 0.22 μm filters (MilliporeSigma™, Burlington, MA, USA, Z359904) prior to ICP-OES measurement. Gold concentration was determined following the same protocol as described in Section 2.4.4.

### 2.6. Statistical Analysis

All statistical analyses and graphs were obtained with GraphPad Prism (version 9.1.0; GraphPad Software, Inc., San Diego, CA, USA). Mean ± s.e.m. values were calculated and plotted in the results. Statistical significance was assessed by two-way analysis of variance (ANOVA) with Tukey’s multiple comparisons test as the post-hoc test method, and the multiple unpaired *t* test.

## 3. Results

### 3.1. GNP Characterization

**Figure 1:**
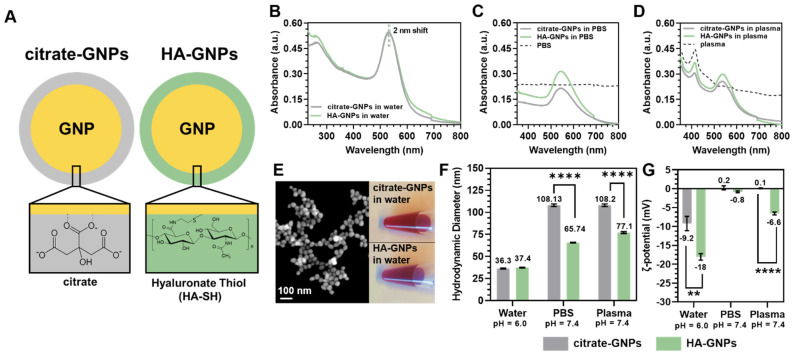
(**A**) Schematic diagram of GNPs stabilized with citrate or surface passivated with thiol-modified hyaluronic acid (where the number of monomers *n* is ~3). Absorbance spectra of citrate-GNPs (gray line) and HA-GNPs (green line) dispersed in (**B**) water, (**C**) PBS, and (**D**) plasma. (**E**) SEM image of the particles after synthesis (130,000×) and optical photographs of citrate-GNPs and HA-GNPs. Measurements of (**F**) hydrodynamic diameter and (**G**) ζ-potential of citrate-GNPs and HA-GNPs dispersed in Milli-Q water, PBS, and porcine plasma. Significance is calculated using a multiple unpaired *t* test (** *p* < 0.005, **** *p* < 0.0001).

In this study, we synthesized spherical GNPs with the citrate reduction method and surface passivated them with hyaluronate-thiol. We previously demonstrated that nanoparticles produced using this synthesis protocol generate a strong surface plasmon resonance peak and prominent X-ray attenuation, useful in nanotheranostic applications [25]. Surface plasmon resonances occur in metal nanostructures at frequencies governed by the material’s properties and geometry [19,67,68,69,70,71], and spectral shifts can be indicative of changes in the local refractive index, such as the presence of a nearby molecule [72]. The schematic in Figure 1A illustrates the molecular interaction and surface chemistry of GNPs stabilized with citrate or thiol-modified hyaluronic acid. Figure 1B shows the absorbance spectra of the citrate-GNPs and HA-GNPs in water at pH 6.0. There is an observed 2 nm red-shift in the SPR peak (from 530 nm to 532 nm) of the GNPs due to a change in refractive index when the particles are passivated with thiol-modified hyaluronic acid. When the solvent of the media is changed to either PBS (Figure 1C) or plasma (Figure 1D), both of which are at a pH of 7.4 and above the pKa values of citric acid (pKa_1_ = 3.13, pKa_2_ = 4.76, and pKa_3_ = 6.39) [73], the intensity of the single particle plasmon at ~530 nm decreases, evident of signs of aggregation and particle instability. Interestingly, in both solvents (PBS and plasma), the HA-GNPs show improved stability over the nascent particles indicated by the higher absorbance values around 530 nm. Figure 1E depicts a SEM image, representative of the particles after synthesis, demonstrating their spherical morphology, as well as photographs of the prepared solutions, highlighting their uniform dark red color. The similarity in red color for both surface functionalizations in water at pH 6.0 is indicative of their colloidal stability at this condition as no sample macro-aggregation or flocculation was observed due to passivation. To assess the anti-biofouling properties of the HA-GNPs and compare them with the citrate-GNPs in a simulated physiological environment, changes in hydrodynamic diameter as well as surface charge of the particles were measured after resuspending them in either water, PBS, or plasma. Rather than using SEM, which can only provide an estimation of the projected area of the particle, DLS offers information of the hydrodynamic diameter of the inorganic core along with any coating material and solvent layer attached to the particle as it moves under the influence of Brownian motion. Therefore, we present DLS measurements rather than estimate size using electron microscopy since it can be obtained in solution and provides a deeper understanding of the surface chemistry, which is crucial for optimizing GNP performance in biological assays and to predict particle migration and biodistribution [74]. As demonstrated in Figure 1F, the hydrodynamic diameter of the citrate-GNPs and HA-GNPs does not significantly differ when dispersed in water at pH 6.0, although the average hydrodynamic size of HA-GNPs increases slightly as a result of the HA coating. However, when immersed in a media with properties similar to that of a biological environment (either PBS for physiological pH or blood plasma as source of physiological pH and proteins), both the citrate-GNPs and HA-GNPs increase in size when compared to that obtained in Milli-Q water. Significant differences are highlighted (*t* test unpaired) between the citrate-GNPs and HA-GNPs dispersed in PBS and plasma (**** *p* < 0.0005). Importantly, the increase in hydrodynamic diameter for the HA-GNPs in both media is significantly less than that of the nascent particles, which is consistent with the absorbance spectra in Figure 1C,D and indicative of particle clustering in the slightly basic media. Furthermore, the fact that the HA-GNPs do not show such drastic changes in diameter is evidence that the HA coating is shielding the particles and providing anti-biofouling properties. For the citrate-GNPs, the average polydispersity indices were 0.32 (water), 0.23 (PBS), and 0.54 (plasma), while for the HA-GNPs, the average of polydispersity indices were 0.32 (water), 0.21 (PBS), and 0.57 (plasma). The increase in polydispersity in PBS and plasma for the nascent particles is also indicative of particle clustering due to instability. Zeta potential measurements can also provide insights into particle stability. The zeta potential of the citrate-GNPs and the HA-GNPs dispersed in Milli-Q water, PBS, and plasma are shown in Figure 1F. A significant increase (** *p* < 0.005) between the absolute value of the zeta potential for the citrate-GNPs and HA-GNPs in water is notable, whereby the charge on the citrate-GNPs is ~50% less negative. The zeta potential changes radically when both particle types are immersed in slightly basic media. However, while the HA-GNP surface charge remains negative in both PBS and plasma (showing less of a change in charge of the particles), the citrate-GNP surface becomes slightly positive, corroborating the spectroscopic findings of particle instability in these media. According to our hypothesis, the molecules and proteins which populate these biologically relevant media, in the case of citrate-GNPs, displace and disrupt the citrate-shell, which charge stabilizes the particles and prevents them from aggregating. We also measured the sizes and surface charges of the particles as a function of time (after 6 h and 24 h of incubation in PBS or plasma) finding no differences compared to the measurements reported in Figure 1F,G. 

### 3.2. In Vitro Cytotoxicity Assessments

**Figure 2:**
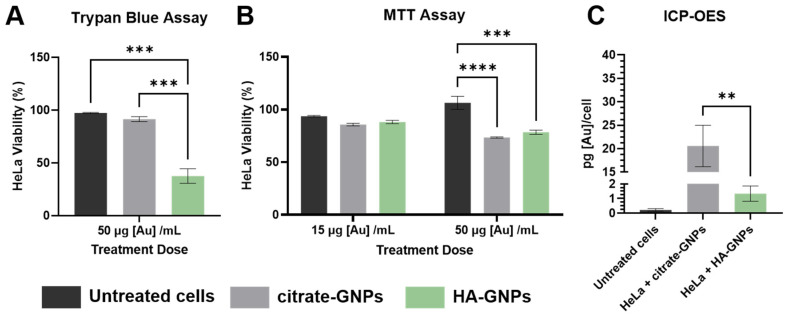
In vitro uptake of GNPs in HeLa cells after a 24h incubation. Cytotoxicity assays (where cell viability is expressed as % of living cells divided by the total cells counted) using (**A**) Trypan blue staining (50 μg/mL per well) and (**B**) MTT (15 μg/mL per well and 50 μg/mL per well) after treatment with citrate-GNPs and HA-GNPs. (**C**) Elemental Analysis performed with ICP-OES on HeLa cell pellets 24 h after treatment with citrate-GNPs and HA-GNPs. A two-way ANOVA test was performed to compare the interactions between each group (** *p* < 0.005, *** *p* < 0.001, **** *p* < 0.0001, Tukey’s multiple comparisons test).

In vitro analyses involving HeLa cells were performed to assess any cytotoxic effects due to the GNP treatments. After 24 h of GNP incubation, cell viability, measured by Trypan blue assay, showed that a high dose concentration of HA-GNPs (50 μg [Au]/mL per well) significantly reduced cell viability by almost 40% (*** *p* < 0.0005) when compared to no treatment or treatment with citrate-GNPs, as shown in Figure 2A. However, it must be considered that despite the fact that dye exclusion assays, such as Trypan blue, offer an easy and rapid technique to selectively stain cells, viability is determined solely based on cell membrane integrity, without considering any information regarding capacity to grow or cell functionality. In fact, it has been proven that even though the cell membrane integrity has been compromised, there is a possibility for the cell to self-repair and become fully viable, affecting the results [75]. Another potential problem with this assay is related to the dye uptake and limits of signal detection which assess viability in a binary way: if a cell is partially disrupted with only a small amount of dye uptaken and the signal is below the limit of detection, the cell will be classified as viable. Therefore, the use of another complementary assay for viability is also recommended to assess cytotoxicity due to treatment. After 24 h of GNP incubation, cell viability measured by MTT assay showed that a moderate concentration (15 μg [Au]/mL per well) of citrate-GNPs and HA-GNPs were not cytotoxic for the cells. However, high dose concentration (50 μg [Au]/mL per well) reduced cell viability by 22% in case of HA-GNPs (*** *p* < 0.001) and almost 28% in the case of citrate-GNPs (**** *p* < 0.0001), as shown in Figure 2B. This data indicates that treatment dose may be a more important factor on the effects of cell viability over particle surface chemistry. Elemental analysis performed with ICP-OES is reported in Figure 2C. The amount of gold is normalized per cell. The amount of gold content quantified after citrate-GNP treatment was significantly higher than that of HA-GNP treatment in HeLa cells: treatment with citrate-GNPs yielded ~20% more gold content than treatment with HA-GNPs after a 24 h incubation period (** *p* < 0.005). This result suggests that the presence of the HA coating shields the particles from the formation of a protein corona which can occur on nascent particles dispersed in a biological media, mediating their interaction and uptake with cells. These results are similar to that reported by Karakocak et al. [76] where nascent GNPs < 40 nm administered to retinal pigment epithelial cells (ARPE-19) showed greater cellular internalization than HA coated GNPs as evaluated with ICP-MS.

### 3.3. In Vivo Biodistribution in a Murine Lung Cancer Model

**Figure 3:**
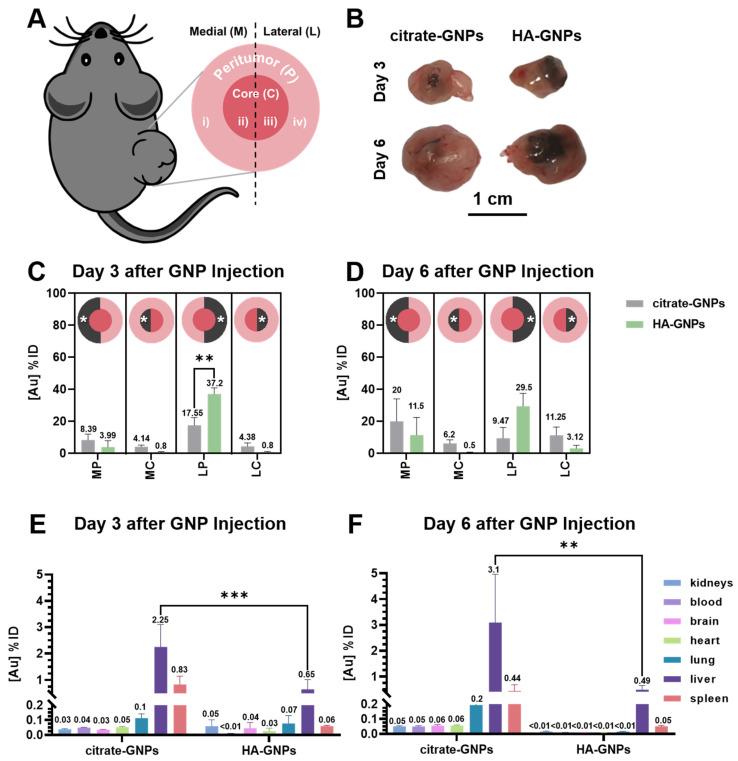
In vivo assessments and quantification of GNP distribution in a murine NSCLC model. (**A**) Schematic of tumor growth and atlas reference for the ex vivo analysis. Tumors were divided in medial and lateral halves (in reference to the mouse body positioning) and each half was subdivided into peritumor and core using a surgical blade. (**B**) Photos of ex vivo LLC tumors taken three and six days after intratumoral injection of citrate-GNPs and HA-GNPs. Tumors were photographed exposing only the lateral side, which allows for better appreciation of the GNP distribution with gross inspection (black areas represent particle clusters). Under the same experimental conditions, HA-GNPs distribute site-specifically in the lateral side of the tumor, while citrate-GNPs remain close to the injection site (in the center of the tumor). Elemental analysis performed using ICP-OES on resected tumors (*n* = 4/group) allow for site-specific comparisons between the citrate-GNPs and HA-GNPs (**C**) three days or (**D**) six days post GNP injection. Biodistribution of GNPs in organs calculated using ICP-OES, where gold concentration in each organ is quantified as percentage of injected dose of GNP (% ID) (**E**) three days or (**F**) six days post GNP injection. A two-way ANOVA test was performed to compare the interactions between each group (** *p* < 0.005, *** *p* < 0.001, Tukey’s multiple comparisons test).

Figure 3A presents a schematic of the approach used in this study to investigate differences in GNP accumulation across different regions of the tumor depending on particle surface chemistry. We chose to subdivide the tumors into four different groups which included differentiating between the medial and lateral side of the tumor because we are not only consistently applying an external force to inject the particles toward the medial side but also because of differences in interstitial fluid pressure (IFP) across the tumor. In fact, Stapleton et al. previously demonstrated that the heterogeneity of the IFP across the whole tumor volume affects the intra-tumoral distribution of CT-liposomes in metastatic breast adenocarcinoma-bearing mice [77,78]. Moreover, spatial measurements of permeability, perfusion, interstitial volume fraction, and plasma volume fraction, revealed heterogeneous changes between the medial and lateral side of the tumor, although this quantification was not thoroughly investigated. Three and six days after intratumoral treatment with either citrate or HA passivated GNPs, mice harboring lung cancer tumors were sacrificed and their tumors resected. Flash frozen tumors were then divided into medial and lateral halves (in reference to the mouse body positioning) and each half was subsequently subdivided into peritumor and core using a surgical blade as described in Section 2.5.3. Figure 3B shows example photos taken from the lateral side of the harvested ex vivo tumors. Differences in the distribution of the particles between the two GNP treatment types are macroscopically evident (particles are observed as dark spots in the tumor). The HA-GNPs distribute site-specifically along the lateral side of the tumor, while the citrate-GNPs present a low diffusion pattern, remaining close to the injection site (in the core of the tumor) for all six days. These observations were also confirmed by elemental analysis performed using ICP-OES on resected tumors. As shown in Figure 3C, 3 days post-GNP injection, almost 50% of the HA-GNPs injected remained present in the tumor, with preferential accumulation in the lateral periphery (LP). Gold quantified in this site-specific area was significantly higher (** *p* < 0.005) in the HA-GNP treated mice when compared with citrate-GNP administration. When compared with the data obtained three days post GNP injection, the six-day data shows a ~10% decrease in accumulation in the LP region for both particle types, while particle accumulation in the medial periphery (MP) region increases. This data suggests that over time and as the tumor grows, both particle types diffuse more across the tumor but are still retained intratumorally. Evaluation of the particle biodistribution using ICP-OES in other organs (liver, spleen, lung, heart, blood, kidneys, and brain) for the two different particle surface passivations are reported in Figure 3E,F. An ANOVA test revealed that the gold content in the citrate-GNP group was significantly higher than that in the HA-GNP group in the livers examined for the data obtained three days post injection, and six days post injection (** *p* < 0.005, *** *p* < 0.001). Notably, for the six-day timepoint, citrate-GNP accumulation in the liver was six times greater than that of the HA-GNP. These results suggest that clearance of the citrate-GNPs from the tumor to other organs is higher for both follow up timepoints, and that HA-GNPs are better retained in the TME, possibly due to receptor-mediated interactions or interactions with the extracellular matrix. Further, in comparing the six-day post-injection data with the three-day post-injection data, the majority of the organs showed an increase in citrate-GNP content and a decrease in the HA-GNP, indicative of their retention in the TME. 

## 4. Discussion

In this work, we fabricated and characterized citrate-GNPs, surface passivated with hyaluronate-thiol, and assessed their in vitro cytotoxicity as well as cellular uptake in HeLa cells. We tested the colloidal stability of the particles as well as their antifouling properties using PBS and plasma to mimic biological media. We noticed that the increase in hydrodynamic diameter for the HA-GNPs in both media was significantly less than that of the nascent particles, proving that the HA coating was shielding the particles and providing anti-biofouling properties. These findings, along with optical spectroscopy and evaluations of surface charge, also suggest that the presence of HA on the surface of GNPs helps to maintain stability of the particles in biological media.

Metal nanoparticle surface modifications with HA have been used in previous studies as biosensors to measure enzymatic activity of hyaluronidase [79,80] and to evaluate their ability to target cancer cells via HA receptors as well as their efficiency in releasing drug cargo at a target site [81]. For instance, Kumar et al. loaded metformin (an antihyperglycemic agent commonly used for the treatment of diabetes also known to reduce the risk of developing hepatocellular carcinoma) on HA capped GNPs, and the authors demonstrated that administration of this formulation exhibited increased cytotoxic activity over free drug in liver cancer cells in vitro without hindering zebrafish embryo development in vivo [82]. However, the authors did not report any information regarding biodistribution of the particles in vivo, an important parameter for the clinical translation of this nanoplatform. While a recent study by Xu et al. [83] did investigate pharmacokinetics and particle biodistribution, this was performed for hyaluronic acid-gold nanorods (HA-GNRs) administered intravenously into nude mice with xenograft MCF-7 breast cancer tumors. The authors showed that 24 h after administration, the majority of particles distributed to the liver (~50%) while <15% were present in the tumors. Such a low percent of the administrated dose reaching the tumor target not only reduces the possibility of particle retention in the tumor over time but also decreases the efficiency of light-based therapy. Our study overcomes these limitations by administering the particles locally into the tumor, where we can monitor their retention over time and quantify site-specific accumulation across different regions of the tumor. Furthermore, in our biodistribution study, we showed that, post-intratumoral injection, there is <1% particle accumulation in the off-target organs assessed, and this percentage does not increase over time.

In this study we used intratumoral injections of GNPs, since local administration has shown superior efficacy compared with systemic administration of nanomedicines, which limits the penetration of therapeutics from the circulation into solid tumors [84]. In addition, in combining these particles with immunotherapeutic agents, a local delivery approach would introduce several advantages: (1) higher concentrations of agents to target antigens and tumor-infiltrating immune cells; (2) lower overall doses, reducing systemic exposure and toxicity; and (3) new possibilities to treat patients with metastases by exploiting the abscopal effect, providing local adjuvant activity to turn malignant tissue into an in situ cancer vaccine.

Although beyond the scope of this work, there are some other interesting aspects of HA that should be discussed for future study. In the human body, HA is typically present at a very high molecular weight (20 MDa) providing elasticity to the tissues [85]. Kuehl et al. [86] tested different molecular weights of iodine-125 labeled HA molecules in the lungs of mice, quantifying organ radioactivity over time after intratracheal instillation. The study demonstrated that molecular weights of HA between 7–67 kDa present rapid systemic distribution, while HA with molecular weights between 67–215 kDa persist longer in the lungs, and HA with molecular weights >215 kDa penetrate poorly to the lungs. Therefore, it may be possible that different molecular weights of HA have an effect on GNP distribution when HA is used as a surface coating. In our study, we used HA with a molecular weight of 10 kDa, but further investigations are necessary to clarify the role of molecular weight of HA and how it can influence diffusion of the metal nanocomplexes in cancerous tissues and how exogenously administered HA can interact with endogenously present HA in the extracellular matrix. 

Another point of discussion is how the presence of HA influences particle uptake by cells as well as particle distribution in tissue in relation to expression of its cell surface receptors, such as CD44, hyaluronan-mediated motility receptor (HMMR), and intercellular adhesion molecule-1 (ICAM-1) [87]. Shen et al. [88] demonstrated that hyaluronic acid functionalized lipid nanoparticles loaded with paclitaxel showed more efficient uptake over lipid nanoparticles without HA in B16F10 melanoma cells, facilitating delivery of the drug into these CD44-overexpressing cancer cells. Chiesa et al. [89] observed that the uptake of hyaluronic acid-chitosan nanoparticles by human mesenchymal stem cells occurs through confinement in cytoplasmic vesicle-like regions ~1–3 µm in diameter, located near the CD44+ membrane surface, also suggesting an endocytosis mechanism for cellular uptake of these particles. However, these previous studies were conducted with non-metallic nanocomplexes. In our investigation, we evaluate the in vitro cytotoxicity as well as cellular uptake in HeLa cells, which are known to express CD44 [90], but further research is required to clarify the mechanisms of interaction between hyaluronic acid passivated gold nanoparticles and HA cell surface receptors.

## 5. Conclusions

In conclusion, in this study we fabricated GNPs and passivated their surface with hyaluronate-thiol using an easy and reproducible method. We investigated their physical characteristics and biofouling properties in biological media and examined any cytotoxic effects in vitro using HeLa cells. Finally, we quantified the site-specific distribution of nascent and HA-GNPs after intratumoral administration in a murine model of lung cancer. Our results support the hypothesis that HA enhances GNP distribution in the peritumoral region. This finding improves our knowledge regarding how changes in the surface chemistry of nanocomplexes can result in different localization of the agent across solid tumors, opening new possibilities for applications of nanomedicine in cancer theranostics.

## Supplementary Materials

The following are available online at https://www.mdpi.com/article/10.3390/biomedicines9111561/s1, Figure S1: Mice weight and tumor manual measurements with the caliper.

## Abbreviations

ANOVA	analysis of variance
DLS	dynamic light scattering
FBS	fetal bovine serum
GNP	gold nanoparticle
HA-SH	hyaluronate-thiol
IACUC	Institutional Animal Care and Use Committee
ICP-OES	Inductively Coupled Plasma-Optical Emission Spectrometry
IFP	interstitial fluid pressure
IT	intra-tumoral
LLC	Lewis lung carcinoma
MTT	(3-(4,5-dimethylthiazolyl-2)-2,5-diphenyltetrazolium bromide)
NSCLC	non-small cell lung cancer
PBS	phosphate buffered saline
PDI	polydispersity index
s.e.m.	standard error of the mean
SPR	surface plasmon resonance
SEM	scanning electron microscopy
TME	tumor microenvironment
