# Supplementary material for: Hyaluronate-Thiol Passivation Enhances Gold Nanoparticle Peritumoral Distribution When Administered Intratumorally in Lung Cancer
Source: Biomedicines. 2021 Oct 28;9(11):1561. doi: 10.3390/biomedicines9111561 (PMC8615404; doi:10.3390/biomedicines9111561)
Supplement: Supplementary file 1 [file biomedicines-09-01561-s001.zip › biomedicines-1422507-supplementary.pdf]

Supplementary Material

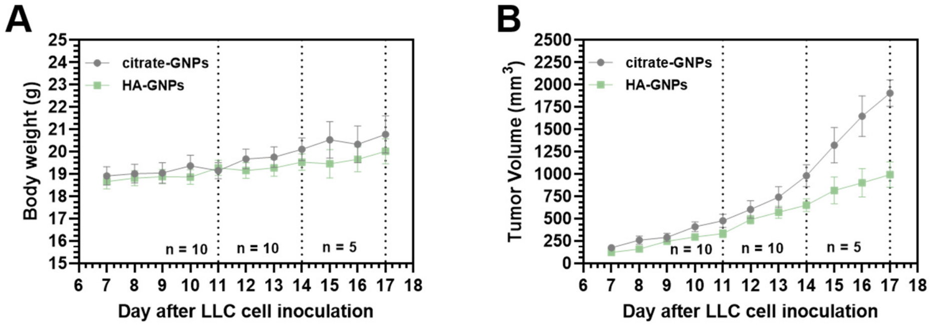

Figure S1. (A) Mice weight and (B) tumor volume (based on manual measurements performed using a caliper for n = 10/group up to 14 days after LLC cell inoculation and n = 5/group after day 14). Dashed lines represent particle injection (day 11), tumor harvest group 1 (day 14), and tumor harvest group 2 (day 17).
